# Examining the influence of knowledge transfer and dynamic capabilities on enterprise digital transformation

**DOI:** 10.1371/journal.pone.0311176

**Published:** 2024-12-16

**Authors:** Xiaofang Xiong

**Affiliations:** Suan Sunandha Rajabhat University, Bangkok, Thailand; Kalasalingam Academy of Research and Education, INDIA

## Abstract

Digital transformation is a crucial strategy for enterprises to navigate the digital economy, leading to a growing number of organizations actively engaging in this process. However, the high failure rates in digital transformation initiatives have pushed both academia and industry to identify effective approaches. Key challenges include a lack of relevant knowledge and capabilities as well as the need for coordinated resource utilization. Knowledge is vital for developing new business models, selecting appropriate digital technologies, and strategizing their use; knowledge transfer is a central component of this transformation. To address these challenges, this study reviews the literature on knowledge transfer, dynamic capabilities, and digital transformation and proposes a theoretical model that connects these elements. Empirical research through questionnaire surveys and structural equation modeling reveals that knowledge transfer significantly enhances digital transformation and positively impacts enterprises’ ability to perceive, capture, and reconstruct knowledge. These abilities also serve as partial mediators between knowledge transfers and digital transformations. This study innovatively integrates knowledge transfer and digital transformation into a unified model, thereby contributing to the theoretical exploration of digital transformation pathways. Moreover, it enhances the understanding of dynamic capabilities in this context, providing valuable insights and practical implications for Chinese enterprises aiming to improve their digital transformation efforts through effective knowledge transfer and cultivation of dynamic capabilities.

## 1 Introduction

Digitization affects every aspect of human society’s production and lifestyle, and the digital economy has emerged as a crucial driving force for global economic development and the future trajectory of the global economy. Presently, the digital transformation of Chinese enterprises is still in its nascent stage, with many struggling to effectively execute digital transformation initiatives. The ability of enterprises to navigate successful digital transformations has become a critical focus and urgent concern for academia. Digital transformation represents a comprehensive overhaul within an enterprise, encompassing not only the adoption and utilization of digital technologies to enhance business processes, but also incorporating changes in organizational structure, business models, value creation methods, and management approaches [1]. Enterprises undergoing digital transformation are confronted with a dynamic and evolving external environment and internal transformation challenges, disrupting their previous stable operational state. Existing knowledge and technologies are often insufficient for addressing the demands of digital transformation. Digital technologies play a pivotal role in supporting new business models within enterprises and driving holistic organizational changes [2]. Traditional enterprises lacking digital technologies, expertise, skilled personnel, and relevant transformation experience risk fall into patterns of dependency on outdated models, hindering their ability to transition and evolve effectively. Enterprise digital platform applications have emerged as a response to these challenges. These digital platforms aggregate vast amounts of operational data, facilitating rapid knowledge transfer and dissemination among users [3]. By offering digital technology services, infrastructure, consulting, and operational solutions, digital platforms assist transitioning enterprises in acquiring digital technologies and knowledge, serving as a crucial conduit for knowledge transfer. The advent of digital platforms streamlines knowledge transfer processes and reduces barriers to entry for enterprises embarking on digital transformation journeys.

The rapid evolution of digital technologies has expedited the rate of change, resulting in a volatile, complex, and uncertain external environment for enterprises. Against this backdrop, scholars, both domestically and internationally, have increasingly recognized the significance of dynamic capabilities in facilitating enterprises’ digital transformation efforts. A substantial body of scholars has conducted research to explore avenues for digital transformation by leveraging the dynamic capabilities theory framework [4]. The enhancement of dynamic capabilities within enterprises constitutes a key challenge to successful digital transformation. Dynamic capability denotes an enterprise’s capacity to adapt to a swiftly changing environment, achieved through the integration, construction, and reconfiguration of internal and external resources and capabilities, ultimately mitigating resistance to change within the organization [5]. The proposition that firms must enhance their dynamic capabilities to identify and seize opportunities during digital transformation is based on established theories of dynamic capabilities, which suggest that organizations need to continually adapt and reconfigure their resources to respond to changing environments [6]. Specifically, we refer to previous studies that demonstrate the link between dynamic capabilities and performance in the context of digital transformation [7–9]. Digital transformation implies that organizations need to search for and apply digital resources external to the organization, and that the knowledge and management experience of the organization is no longer sufficient for digital transformation. For example, Smith and Johnson found that firms that can proactively identify and integrate external digital resources show higher innovation and market adaptability than those that rely on internal resources. This study suggests that a firm’s ability to utilize external digital resources during the digital transformation process directly affects the degree of its transformation success [10]. Failure to acquire digital knowledge and best practices in digital transformation from external sources can impede the development of dynamic knowledge-based capabilities that are critical for successful digital transformation [11].

In the digital economy era, enterprises are actively seeking to integrate digital technologies and unearth new avenues for growth and development. Many enterprises prioritize digital transformation as a key strategic imperative in response to this evolving landscape [10]. Consequently, the realization of digital transformation pathways has emerged as a focal point of academic research. For the purposes of this study, ‘realization’ is defined as the process by which an enterprise recognizes the need for and importance of digital transformation. This stage involves the firm’s perception of changes in the external environment and understanding of the gap between its current capabilities and future needs. It represents a thought process–that is, the perception and understanding of the need for change. Although scholars have explored the path of digital transformation and the importance of digital knowledge, relatively few studies have been conducted on knowledge transfer behaviors in the digital transformation process of enterprises. Previous studies have mainly focused on the impact of dynamic capabilities on digital transformation or the enhancement of dynamic capabilities by knowledge transfer, and lack in-depth analysis of how knowledge transfer behaviors affect digital transformation by enhancing the dynamic capabilities of enterprises [12]. Understanding the relationship between knowledge transfer and dynamic capabilities and the implications of this relationship for digital transformation, therefore, becomes an important entry point for this study.

Combining a literature review and field research, we find that enterprises are often not bound to a specific way when acquiring knowledge related to digital transformation and that knowledge transfer activities are more often characterized by knowledge acquisition and knowledge application, which is determined by the nature of the transferred knowledge itself. This study validates the facilitating effect of knowledge transfer on digital transformation and constructs a new theoretical model of knowledge transfer that promotes enterprise digital transformation by driving dynamic capabilities. By empirically testing the validity of the model, this study highlights the importance of knowledge resources in the process of dynamic enterprise capability construction and digital transformation.

This study considers the digital transformation of enterprises as the research object and focuses on the key role of ‘innovation performance’ in this process. The literature on knowledge transfer and dynamic capabilities is reviewed, and dynamic capabilities are subdivided into sensing capabilities, capturing capabilities and reconfiguring capabilities, and a theoretical model of ‘knowledge transfer-dynamic capabilities-digital transformation’ is constructed. This study aims to investigate how knowledge transfer affects the digital transformation of enterprises and reveals the paths for enterprises to cultivate dynamic capabilities through knowledge acquisition and application to improve innovation performance and achieve digital transformation. This study not only provides new perspectives for relevant theoretical innovations but also provides important references for enterprises to enhance innovation performance more effectively in digital transformation practice.

## 2 Literature review

### 2.1 Knowledge transfer

Knowledge transfer is a fundamental aspect of knowledge management that encompasses the transmission of knowledge across different domains of expertise. Wehn and Uta posit that knowledge transfer involves actively sharing one’s own knowledge with others or engaging in consultations to exchange knowledge [13]. As highlighted by Linde et al., knowledge transfer plays a crucial role in addressing technological challenges and operational issues within organizations by absorbing and effectively utilizing the transferred knowledge [14]. While discussions of “knowledge transfer” often reference “knowledge sharing,” scholars underscore that knowledge sharing typically occurs at an individual level within organizations, whereas knowledge transfer extends beyond this scope to encompass knowledge exchange at higher levels such as among groups, product lines, or departments [15]. Peng contend that knowledge transfer not only facilitates knowledge sharing but also optimizes firms’ knowledge structures and enhances their innovation capabilities [16].

Many scholars have studied various factors that affect knowledge transfer. The knowledge transfer model they constructed typically includes four dimensions: knowledge, knowledge source, knowledge receptor, and transfer situation [17]. Specifically, the motivation, willingness, ability, and trustworthiness of the subject of knowledge transfer are crucial for facilitating knowledge transfer, whereas the characteristics of the transferred knowledge (e.g., implicitness, embeddedness, complexity, and specialization) may impede the effective transfer of knowledge [18]. In addition, the transfer context and network relationships play complex roles in knowledge transfer. Research has shown that the contextual distance between the knowledge source and the knowledge recipient should be kept moderate, and that too small or too large a distance can reduce the knowledge recipient’s satisfaction with the transferred knowledge [13]. Appio et al. combined cognitive psychology with the organisational context in their research, they emphasized that knowledge transfer at the departmental or organisational level, above the individual level, often involves important social interaction processes such as sharing, interpreting and synthesizing information [19]. These social processes are key to the effective flow and utilization of knowledge. Scholars have generally focused on how knowledge transfer can enhance firms’ competitive advantages and innovation performance [20].

In addition, effective knowledge acquisition and utilisation has been shown to be important for building dynamic capabilities and driving enterprise digital transformation. Li et al found through a case study of industrial internet platform user enterprises that user enterprises drive knowledge trading and reuse through trust in the platform, thereby acquiring and applying digital knowledge to help build digital infrastructure, improve digital management capabilities and facilitate enterprise transformation. The following is a case study of an industrial internet platform user enterprise [2]. Hu et al. show that the knowledge distance between SMEs and professional service organisations negatively affects firms ‘digital transformation performance, and that firms’ collaborative capabilities can reduce this knowledge distance and improve the effectiveness of digital transformation [4]. Knowledge transfer can promote firms’ dynamic capabilities, and a model was constructed to show that knowledge transfer improves the performance of new service development by enhancing firms’ dynamic capabilities.

In general, although scholars do not make a strict distinction between ‘knowledge transfer’ and sharing in real-life research, they usually involve different levels of knowledge exchange processes. Knowledge transfer should not be seen as a unidirectional transfer of knowledge, but rather as a two-way interactive process in which the knowledge transfer agent (e.g., an organization or an employee) identifies important knowledge that is needed, uses knowledge transfer mechanisms to acquire the knowledge, and then effectively disseminates it to the relevant people by continuously integrating old and new knowledge. By making innovative use of this knowledge, firms are able to increase their knowledge stock and thus enhance their innovation performance [11].

### 2.2 Dynamic capabilities

Teece introduced the concept of dynamic capabilities in 1997, highlighting the significance of an organization’s ability to adapt and modify its internal processes and practices in response to a rapidly changing environment. Teece argues that a firm’s competitive advantage hinges on its capacity to consistently innovate and deliver new products and services to customers in a dynamic market landscape [6].

The 3P framework of factors influencing dynamic capabilities developed by Teece et al. provides a clear structure for understanding dynamic capabilities consisting of the following three main dimensions:

- Positioning

Positioning theory is based on the resource-based view and the positioning school of thought and refers to the arrangement of a firm’s resource portfolio, structure, and resource stock. It is divided into two parts: internal and external parts. Internal positioning focuses on the unique technology, reputation, intellectual property, organisational structure and customer base of the enterprise, while external positioning focuses on the impact of external factors such as industry structure, competitive environment and market position on the enterprise [21].

- Path

Path involves the historical process of a firm’s development, including path dependence, business practices, organizational learning, and technological opportunities. Path dependency theory states that the historical direction of a firm’s development influences current and future decisions and behaviors, which emphasizes the importance of past experience in shaping a firm’s capabilities [22].

- Process

A process is a core element of dynamic capabilities and involves the processes of coordination and integration, learning, innovation, and reconfiguration within an organization. This includes internal coordination (integration of different functions, resources, and business activities) and external coordination (e.g., relationships with partners, customer management, and strategic alliances). Learning capability is part of a firm’s core competence, enabling it to adapt to changes in the external environment and to develop strategies accordingly. Innovation and restructuring are important ways for firms to respond to environmental changes, including asset restructuring, process renewal, and capability reengineering [6].

Within this theoretical Structure Knowledge framework, this study explores how digital empowerment enhances firms’ dynamic capabilities through knowledge transfer, thereby improving innovation performance. The need for dynamic capacity is further supported by the resource-based view [23]. According to this concept, the unique and scarce resources within a firm can create a lasting competitive advantage. These resources must be continually integrated and reconfigured to adapt to changing market conditions [9]. By analyzing practical examples (e.g., the successes of companies such as Apple and Tesla), we can clearly understand the key role of resources in the link between dynamic capabilities and innovation performance [18].

The concept of dynamic capabilities emphasizes that a firm’s competitive advantage depends on its ability to update and change its internal processes and practices to continuously deliver innovative products and services to its customers. In a dynamic environment, the traditional view of the resource base alone can no longer sustain competitive advantage, and firms must remain competitive in the marketplace by developing strong dynamic capabilities [6]. Dynamic capabilities allow firms to respond flexibly to changes in market conditions and continuously gain and maintain competitive advantage [20]. The academic classification of dynamic capabilities is largely based on Teece’s research and is usually categorized into three dimensions: sensing, capturing, and reconfiguring. These capabilities help companies identify market opportunities, innovate products and services, and reconfigure organizational assets to meet new demands [6].

In a study by Feroz et al. on the influence of big data resources on digital transformation performance, mediated by digital dynamic capabilities, these capabilities were classified as digital perception, digital exploitation, and digital reconfiguration capabilities [24]. Wu et al. explored the role of dynamic capabilities in advancing enterprise digital transformation by categorizing them as external and internal dynamic capabilities [9]. External dynamic capabilities assist in identifying and creating opportunities, encompassing the capacity to navigate the external environment by perceiving, capturing, and transforming the capabilities. Internal dynamic capabilities focus on integrating internal and external resources into an organization’s structure.

In summary, the consensus among scholars in the study of dynamic capabilities includes the following: on the one hand, enterprises need to continuously develop dynamic capabilities to adapt to rapidly changing environments; on the other hand, the enhancement of dynamic capabilities is realized through the transformation of operational capabilities. Meanwhile, the construction of dynamic capabilities relies on the identification and reorganization of resources to enhance the overall organizational capabilities of enterprises.

### 2.3 Digital transformation

With the introduction of digital technologies, organizations have undergone profound changes in their business processes, process management, and customer value orientation. Research has shown that digital technology significantly improves operational efficiency by optimizing process management, and increases sensitivity to market needs by leveraging advanced market knowledge [4]. Against this backdrop, companies need to adapt to the digital changes that occur in the industry to respond to market demands. The word ‘transformation’ in the term digital transformation emphasizes that this process is not just a change in technology, but requires comprehensive thinking and change at the strategic level. The digital transformation of an organization should focus on the overall strategy, not just functional thinking using digital technology [25]. Rogers contends that digital transformation, involving business model innovation and the optimization of customer needs and experiences, is fundamentally a strategic shift rather than a technological endeavor [26]. The impact of digital technology on business models, organizational structures, and processes is critical for successful digital transformation.

The drivers of digital transformation are a focus of academic research on why and under what circumstances firms undertake digital transformation. According to Peng et al., changes in the market and competitive environment are key drivers of digital transformation for companies [16]. Furthermore, Ghosh et al. state that changes in customer behaviour and expectations are often a direct trigger for digital transformation [5]. With the rapid development of digital advances, companies are facing increasing competitive challenges, and the pressure to digitize is exacerbated by new entrants to disruptive business models and the evolution of the overall technological landscape [1]. The factors driving enterprises’ digital transformation can be analyzed from two main perspectives: macro and micro. The macro perspective focuses on the impact of the external economic environment or policies; for example, Li et al. found that the open-door policy can promote the digital transformation of enterprises [2]. By contrast, the micro perspective focuses on the impact of internal capabilities and behaviors on digital transformation, with Baiyere suggesting that for manufacturing firms, the success of digital transformation depends on the corresponding capabilities of the firm [3].

Recent research has shown that digital transformation is not only the process of applying digital technologies to various production and operational activities within an enterprise but also triggers comprehensive changes in business processes, business models, organizational structures, and management styles. For example, Vial emphasizes that digital transformation covers a profound reengineering of all aspects of business operations, and it requires firms to rethink how they use technology to create value. In implementing digital transformation, firms need not only the application of technology but also a digital mindset, which is a prerequisite for making strategic changes [27]. Singh et al. stated that digital transformation requires firms to shift to a technology-driven strategic mindset and revisit their value chains and market positioning [25]. Furthermore, Rogers highlights the importance of innovating business models and optimizing the customer experience in digital transformation, noting that this is not limited to the use of technology but requires a fundamental shift in the organization’s strategy [26]. To support digital transformation, firms need to build new knowledge systems and capabilities to overcome long-established organizational path dependencies.

Therefore, knowledge transfer behavior is particularly important in this process. Through effective knowledge transfer, firms can update and integrate their knowledge resources, which provides the necessary capability base for digital transformation and fosters dynamic capabilities [11]. Dynamic capabilities are not only key capabilities to cope with changing environments but are also central to driving innovation and achieving long-term competitive advantage. Therefore, this study argues that knowledge transfer behavior plays an important role in facilitating the effects of digital transformation in firms.

### 2.4 Research hypotheses

#### 2.4.1 Knowledge transfer and digital transformation

In the process of digital transformation, knowledge is crucial for firms to understand new consumption patterns, supply, and demand, use knowledge extraction tools effectively, and invest in appropriate digital technology products or services. However, many traditional firms have significant deficiencies in their digital technology and managerial knowledge. Klein, in his SWOT analysis of digital transformation in SMEs in the context of the New Crown Epidemic, identifies a lack of managerial and technological knowledge, as well as a loss of knowledge, as important disadvantages faced by SMEs when undergoing digital transformation. These firms usually lack the expertise to exploit the technological potential of digital transformation, managers have a poor understanding of how to apply digital solutions to business processes, and employees lack the ability to integrate different digital tools effectively, which results in a lack of the skills required to undertake large-scale transformation projects [28].

Further, SMEs tend to focus only on short-term planning, with information technology (IT) management concentrated more at the operational level than at the strategic level. This situation makes enterprises relatively weak in terms of the accumulation of infrastructure, tools, and technologies, thus affecting their ability to acquire and integrate new knowledge [29]. In addition, traditional enterprises have many deficiencies in knowledge management, often managing only tacit knowledge and lacking the systematic management of explicit knowledge. This situation makes enterprises highly dependent on the tacit knowledge of key employees, and the loss of these key employees greatly weakens the knowledge base and competitiveness of enterprises.

In this context, companies’ digital transformation must rely on a rich knowledge base, and traditional companies can acquire the new knowledge they need through knowledge transfer. According to Crupi et al., acquiring new knowledge is an important prerequisite for successful digital transformation of enterprises [30]. Through knowledge transfer, enterprises can access both digital technical expertise and managerial knowledge, along with the practical experience essential for achieving successful digital transformation and the innovative thinking required to drive digital initiatives within the organization [31]. The transfer of knowledge related to digital transformation expands enterprises’ knowledge repository. By leveraging this acquired knowledge internally, enterprises can enhance decision-making capabilities, elevate digital technology and managerial competencies, and effectively propel the enterprise through the digital transformation process.

By highlighting the relationship between knowledge transfer and digital transformation, it is clear that knowledge transfer is the basis for achieving digital transformation. This study proposes the following hypothesis:

H1. knowledge transfer positively affects digital transformation

#### 2.4.2 Knowledge transfer and dynamic capabilities

In today’s highly competitive environment, knowledge transfer can significantly enhance an organization’s core competencies. Organizations that excel at knowledge transfer are better able to survive and thrive in volatile markets than those that are not skilled. This is mainly due to the fact that knowledge transfer increases the flexibility of organisations, thus enhancing the ability of firms to adapt to changes in the external environment, i.e. dynamic capabilities. Knowledge transfer usually occurs when there is a knowledge potential gap between firms, and acquiring external knowledge and creating new knowledge helps firms maintain and enhance their dynamic capabilities [32].

Nowadays, it is often difficult for enterprises to meet the changing needs of their existing knowledge, and creating new knowledge on their own is not only costly, but also has a long lead time, so they cannot respond to rapid changes in the external environment in a timely manner. The main channel for organizations to acquire knowledge is often from external sources, and effective knowledge transfer can significantly increase the amount of knowledge and optimize the knowledge structure of the organization. As the main method to optimize the knowledge structure, knowledge transfer directly affects and enhances the dynamic capabilities of an organization [15]. Access to up-to-date digital information and knowledge enables firms to be more sensitive to changes in digital technology and markets, and thus, effectively integrates resources to adapt to the dynamic external environment. Consequently, knowledge transfer effectively enhances an enterprise’s dynamic capabilities by updating its knowledge structure and stock.

Many scholars have shown that knowledge transfer can, to a certain extent, promote the formation and development of enterprises’ dynamic capabilities. The evolution of dynamic capabilities is based on knowledge; knowledge transfer plays a guiding role in this process. Li et al. pointed out that knowledge sharing provides enterprises with redundant resources and potential for change, which is the basis and key to the development of dynamic capabilities [18]. Because dynamic capabilities are those developed by enterprises to adapt to rapidly changing environments and existing knowledge is often unable to meet such needs, their construction needs to rely on new knowledge resources and need to be continuously updated to reflect environmental changes. Updating the organizational knowledge structure through knowledge transfer is the primary way to build dynamic capabilities. Peng et al. suggested that knowledge transfer positively affects dynamic capabilities through the mediation of organizational learning [16].

In the context of digitization, a firm’s ability to capture resources, process digital information, and reconfigure it is particularly important. Yin and Shi state that capture ability is embodied in the embedding of processed digital information into the activities of the various organisational processes [33]. In contrast, the reconstructive ability of the organization is the basis for continuous strategic design and flexible governance structures that enable the organization to respond in a timely manner to rapidly changing environments [6]. In addition, digital knowledge not only enhances the digital hardware and software of an organization but also fosters its digital management capabilities [34]. Consequently, enterprises can leverage knowledge transfer to reshape their knowledge structure, establish a resource foundation for dynamic capabilities, and foster them to a certain extent.

Based on the aforementioned analysis, this study proposes the following hypotheses:

H2. knowledge transfer positively affects perceived ability
H3. knowledge transfer positively affects capture ability
H4. knowledge transfer positively affects reconstructive ability

#### 2.4.3 Dynamic capabilities and digital transformation

Currently, with the age of rapidly evolving digital technologies, the pace of change in an organization’s external environment has accelerated, resulting in digital transformation facing an environment very different from that of a typical strategic change. It is, therefore, particularly important to have dynamic capabilities to support businesses in responding to these environmental changes, which include the ability to be keenly aware of new digital opportunities and threats, as well as to reconfigure the organization’s resources and capabilities. In this dynamic and unpredictable marketplace, businesses must be flexible, specifically, they need to: (1) allow for frequent changes in organisational roles; (2) respond to changing customer needs and introduce new digital technologies; and (3) respond effectively to increased competition as market boundaries blur and barriers to entry are lowered [35].

Digital transformation presents unique challenges for organizations, which not only need to balance the development of existing functionality but also build new digital functionality that is compatible with past path dependencies [14]. Against this backdrop, digital transformation changes business models, business processes, and organizational structures, and enterprises need dynamic capabilities to cope with rapid changes in technology and markets, as they face the dilemma of blending and converging the old with the new. To achieve digital transformation, enterprises must recombine digital resources with other organizational resources to change their businesses [24].

By leveraging the new digital knowledge gained, dynamic capabilities can help enterprises sense and seize market opportunities brought about by digital technologies so that they can deploy resources to update products, services, or processes, and adjust organizational strategies to balance conflicts that may arise during the transformation process [31]. Specifically, awareness capabilities enable organizations to identify the latest digital trends or new user needs, build the digital skills required to enable digital transformation, and anticipate and respond to changes in user needs. Capture ability enables organizations to quickly identify and respond to potential opportunities and threats by efficiently dispatching resources to seize opportunities and meet demand, which in turn requires organizations to actively build digital capture capabilities to enhance digital agility [9].

In the process of realizing their digital strategies, enterprises not only need to develop sensing and capturing capabilities but also need to reconfigure their capabilities to drive organizational change. In the context of the digital economy, reconfiguration capabilities support the digital transformation of enterprises, which can be strategically driven by building internal and external digital ecological networks and implementing digital architectural changes [31]. The presence of reconfiguration capabilities ensures that organizations are able to sustain strategic change, thereby adapting to a rapidly changing environment and facilitating continuous strategic renewal during digital transformation [8]. Therefore, refactoring capabilities support existing firms in making continuous strategic changes to ensure that the enterprise structure is adapted to a rapidly changing environment, leading to continuous digital strategy renewal during the digital transformation process.

Based on the above analyses, this study proposes the following hypotheses:

H5. Perceived ability positively influences digital transformation
H6. Capture ability positively influences digital transformation
H7. Reconstructive ability positively influences digital transformation

#### 2.4.4 Mediating role of dynamic capabilities

Although hypotheses H5, H6 and H7 reveal the direct impact of dynamic capabilities on digital transformation, these capabilities alone are not sufficient to fully explain the mechanisms by which firms successfully achieve digital transformation. The role of knowledge transfer in this process cannot be ignored. To gain a deeper understanding of how knowledge transfer affects the digital transformation of a firm, mediating variables are introduced: perception capability, capture ability, and reconfiguration capability. These mediating variables act as bridges between knowledge transfer and digital transformation.

Dynamic capability is the ability of an enterprise to continuously adapt to the external environment owing to its complexity and volatility [5]. This capability covers not only the perception of the external environment but also the capture of new opportunities, integration, and reconstruction of resources to improve the competitive advantage of the enterprise. In the process of digital transformation, dynamic capability becomes an important means of coping with changes in the external environment and conflicts within an enterprise. By continuously strengthening dynamic capabilities, enterprises can continuously maintain competitive advantages in digital transformation, thus promoting the in-depth implementation of transformation [36]. Dynamic capabilities enable enterprises to maintain their competitive advantages in digital transformation and thus continuously drive digital transformation.

Knowledge, as an important resource of enterprises, can help enterprises accurately and clearly grasp external market and environmental changes, and many scholars take knowledge resources as the driving force for the formation of dynamic capabilities [37]. Knowledge transfer can enhance dynamic capabilities, and the process of knowledge transfer is a process of dynamic capability evolution. Li et al. pointed out that knowledge transfer enhances dynamic capabilities and that knowledge transfer itself is a process of dynamic capability evolution [2]. Firms with complementary knowledge, experience, and skills are more likely to successfully perceive and capture opportunities as well as reconfigure organizational resources, capabilities, and structures. Thus, firms can enhance their ability to sense and capture knowledge through knowledge transfer in the process of acquiring and applying technological and operational management knowledge.

Specifically, dynamic capabilities alone are not sufficient to fully explain a company’s success in the digital transformation process. Knowledge transfer, as an enabler, plays a crucial role in this process. Therefore, further analysis of the relationship between knowledge transfer and firms’ dynamic capabilities is particularly important for understanding its impact on the digital transformation process. Through knowledge transfer, firms are not only able to acquire information based on changing trends in digital technologies, the degree of digitization of their competitors, and changing trends in the market, but are also able to increase their adaptability to these changes [38]. A higher perceived capacity allows companies to recognize updates in digital technology and management knowledge, which in turn enhances their responsiveness to market opportunities and improves their ability to capture them [32]. This process accelerates the digital transformation of enterprises, enabling them to respond more effectively to cyclical changes in markets and technologies.

Therefore, the ability to reconfigure is another key element in the digital transformation of enterprises. The wealth of knowledge and information provided by knowledge transfer enables enterprises to adapt their organizational structures and processes in a timely manner to the changing market and technological environment [11]. This enhanced capability drives enterprises’ digital transformation and ensures their competitiveness and agility in a dynamic environment. Thus, this study proposes the following hypotheses:

H8. Perceived ability has a mediating effect between knowledge transfer and digital transformation
H9. Capture ability has a mediating effect between knowledge transfer and digital transformation
H10. Reconstructive ability mediates the relationship between knowledge transfer and digital transformation.

### 2.5 Model construction

Based on the research hypotheses of this study, a theoretical model was constructed to explore the relationship between knowledge transfer, dynamic capabilities, and digital transformation. Specifically, knowledge transfer was regarded as the independent variable, dynamic capabilities as the mediating variable, and digital transformation as the dependent variable. The dimensional division of dynamic capabilities is further subdivided into three key dimensions: perceived ability, capturing ability, and reconstruction ability. Perceived ability refers to a firm’s ability to identify and understand external market changes, technological trends, and opportunities. In this model, perceived ability is used as a dimension of dynamic capability to explain how firms can increase their sensitivity and responsiveness to market dynamics through knowledge transfer. Capture ability means that firms can quickly and effectively exploit the opportunities they identify and integrate necessary resources. Knowledge transfer can provide firms with necessary technical and managerial information, which is essential for capturing new opportunities. Therefore, incorporating capture ability into the model can better illustrate how knowledge transfer directly affects the success of digital transformation. Reconstructing ability relates to the flexibility of a firm to adapt its organizational structure, processes, and resources in response to external changes. Knowledge transfer provides firms with advanced best practices and operating models to help them reconfigure their organizations more effectively. By incorporating reconfiguration capabilities into the research model, we can analyze how firms can use knowledge transfers to improve their adaptability and agility in the transformation process.

**Fig 1:**
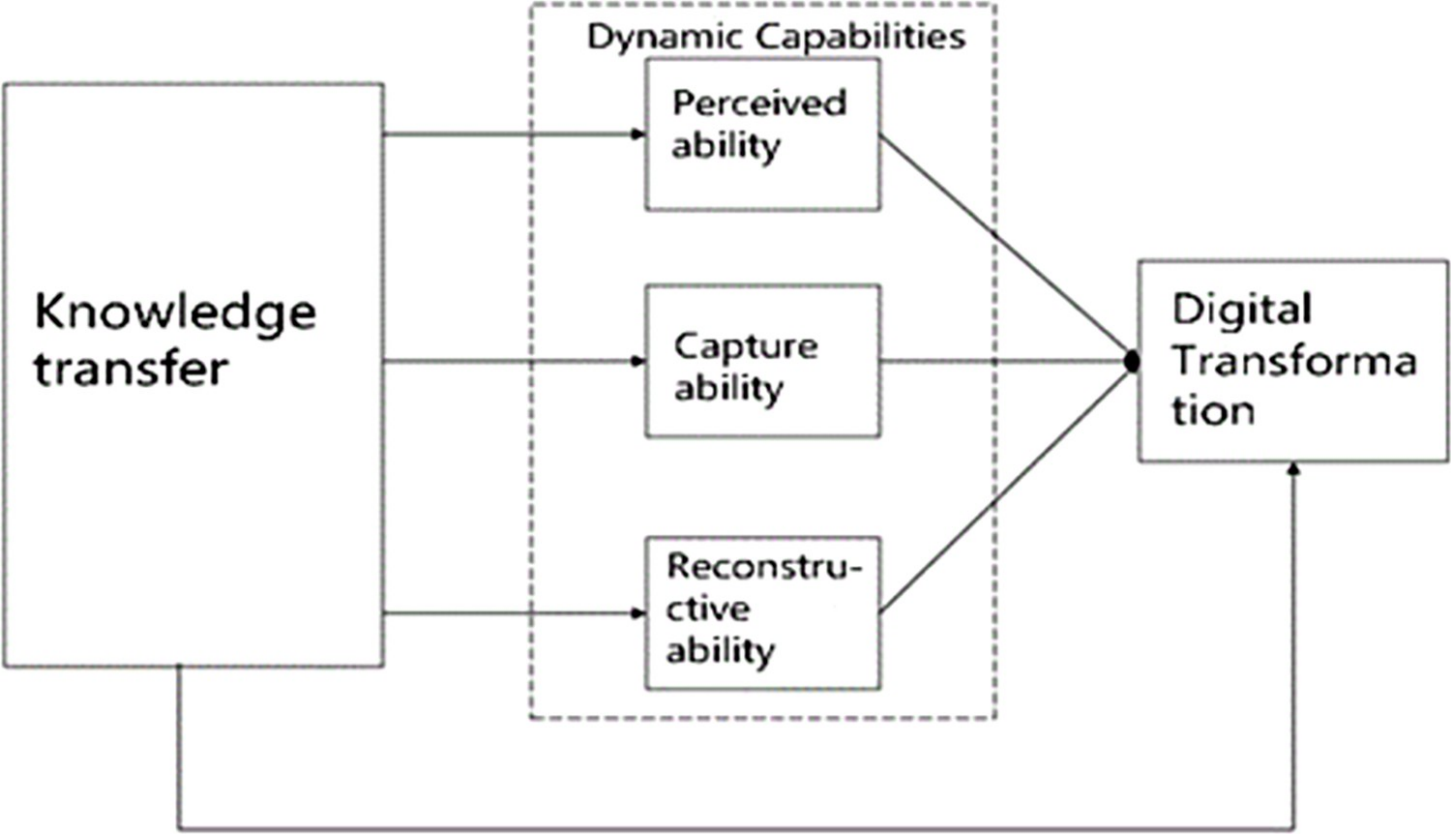
Theoretical model.

This study aims to reveal the interrelationships between these variables. In this model, knowledge transfer not only leads to the enhancement of various dynamic capabilities but also strengthens the competitive position of firms in the digital transformation process through these capabilities. This structured analysis contributes to an in-depth understanding of how firms can achieve success in digital transformation, which provides guidance for related theories and practices. The theoretical model is shown in Fig 1.

## 3 Materials and methods

### 3.1 Duration of the study

The duration of this study spans from the 16th of March 2024 to the 25th of June 2024, marking the specific timeframe for the recruitment period. This period encompasses the initiation of participant recruitment on March 16, 2024, and concludes with the final recruitment date on June 25, 2024. During this period, the researcher engaged in various activities aimed at enrolling eligible participants to ensure a robust sample size for the study. This timeframe was carefully selected to facilitate adequate recruitment while allowing for any necessary adjustments or follow-ups with participants, thereby enhancing the overall effectiveness and reliability of the research outcomes.

### 3.2 Data and sample collection

Data for quantitative research were collected from April 10, 2024, to April 28, 2024. After verifying the validity and reliability of the questionnaire responses, the researcher organized and edited the data. The questionnaire survey served as the primary data source, utilizing established scales recognized in relevant academic fields. Questionnaires were distributed online via platforms and social media, targeting college students to ensure result validity and representativeness, while maintaining anonymity. The survey focused on enterprises undergoing digital transformation, with participation facilitated through communication with middle and senior managers. Of the 223 collected questionnaires, 203 were deemed valid, resulting in a validity rate of 92.59%.

### 3.3 Measurement tools

#### 3.3.1 Measurement of knowledge transfer

In this study, knowledge transfer is defined as the process through which enterprises acquire digital technical and management knowledge from digital platforms or other organizations, including user needs analysis and best practices in digital transformation, and apply this knowledge to practice. Based on the research results and scale design of Huang and Wu et al. [9, 37], this paper specifies “knowledge” is specified as “knowledge related to digital transformation” for measuring the variable of enterprise knowledge transfer. The test data showed a Cronbach’s alpha coefficient of 0.889 for the Knowledge Transfer Scale, indicating its high reliability and suitability for further application.

#### 3.3.2 Measurement of dynamic capabilities

After sorting out the related studies on dynamic capabilities, and taking into account the characteristics of knowledge transfer and digital transformation, we classified dynamic capabilities into three dimensions, namely perceived ability, capture ability and reconstructive ability, by drawing on the studies of Teece [6], Huang [37], and Peng et al. [16]. When setting up the dynamic ability measurement items, the reference scales were collated by referring to the mature scales used in the studies by Huang et al. and which have been empirically tested [9, 37]. The measurement scales for perception ability, capture ability, and reconstruction ability were obtained. The total Cronbach’s alpha coefficient of the mediator variable dynamic capabilities scale was 0.872, and the Cronbach’s ɑ coefficients of the three dimensions were 0.918, 0.885, and 0.871.

#### 3.3.3 Measurement of digital transformation

Scholars assess digital transformation in terms of technology application, strategy, organizational structure, management, teams, and leadership [18]. Wu et al. collated the research lineage of digital transformation-related literature and classified digital transformation into five categories: products, services, processes, models, and organizations [9]. Kamalaldin et al. argued that the outputs of digital transformation of an enterprise include the innovation of products, services and processes [12]. Bringing in the test data from this study yielded a total Cronbach’s alpha coefficient of 0.887 for the dependent variable, Digital Transformation Scale. Therefore, the scale used in this study has a high level of reliability and can be further applied to this study.

### 3.4 Methods of data analysis

Data analysis was conducted using SPSS 24.0, and AMOS 24.0, employing a range of statistical methods. Scale reliability testing was performed using Cronbach’s alpha to evaluate the consistency and reliability of each variable scale. This was followed by scale validity testing to ensure the accuracy of the measurement results and their alignment with the research objectives. Correlation analysis was utilized to assess the relationships between knowledge transfer, dynamic capabilities, and digital transformation, while structural equation modeling was employed to further explore these relationships, validating the study hypotheses through path coefficients and model fit evaluation.

### 3.5 Ethics statement

This Study Approved by Suan Sunandha Rajabhat University Ethics Committee (certificate number: COE. 2-201/2024; 67-024-2-1). Verbal informed consent was obtained from the interviewees and their anonymized information was published throughout this paper. To protect the interviewees, this study adhered to ethical standards of research, and ethical concerns were dynamically achieved throughout the data collection and analysis process.

## 4 Empirical analyses

### 4.1 Reliability testing

The reliability test assessed the internal consistency of a scale, with higher Cronbach’s alpha values indicating greater reliability. A value over 0.7 is typically considered reliable. In this study, SPSS was used to evaluate the reliability of the five variable subscales: knowledge transfer, perceived ability, capture ability, reconstructive ability, and digital transformation, yielding Cronbach’s alpha values of 0.893, 0.891, 0.863, 0.889, and 0.930, respectively, all exceeding 0.8. The overall scale also underwent a reliability test, with a Cronbach’s alpha coefficient of 0.931, indicating excellent reliability. Table 1 presents the results.

**Table 1:** Cronbach’s alpha coefficient of the scale.

Variable	Items	Cronbach’s alpha value after deletion of items	Cronbach’s alpha value
Knowledge transfer	KT1	0.861	0.889
	KT2	0.845	
	KT3	0.872	
	KT4	0.853	
Perceived ability	PA1	0.914	0.918
	PA2	0.864	
	PA3	0.865	
Capture ability	CA1	0.840	0.885
	CA2	0.858	
	CA3	0.813	
Reconstructive ability	RA1	0.813	0.871
	RA2	0.823	
	RA3	0.818	
Digital Transformation	DT1	0.856	0.887
	DT2	0.872	
	DT3	0.854	
	DT4	0.863	
	DT5	0.867	

### 4.2 Validity test

Initially, the KMO and Bartlett’s sphericity tests were conducted on the scale items to assess their suitability for factor analysis. A KMO value closer to 1 indicates higher reliability, with values above 0.7 considered reliable for further research without modifications. Bartlett’s test p-value should be less than 0.05, and values < 0.01 indicate a strong correlation among variables. In this study, the SPSS software produced a KMO value of 0.911, indicating good reliability, and a Bartlett’s test p-value of less than 0.01, confirming significant correlations and the appropriateness of proceeding with factor analysis. Table 2 presents the results of this study.

**Table 2:** KMO and Bartlett spherical test.

**KMO value**		.905
**Bartlett Sphericity Test**	**Approximate chi-square**	2430.745
	**df**	153
	**p-value**	0.000

The results of this test indicate that further exploratory factor analysis can be performed. An exploratory factor analysis of the 18 question items was conducted using SPSS software with principal component analysis to extract the common factors from the items. The results of the study are shown in Table 3, indicating that five male factors could be extracted from the 18 question items. The cumulative explained variance contribution rate of these five male factors reached 77.563% (> 60%), indicating that they can explain the majority of the information on the scale. The degree of explanation for the 18 question items by extracting these five male factors was 77.563%.

**Table 3:** Total variance explained.

Component	Total	Percentage of variance of initial eigenvalues	Cumulative per cent	Total	Percentage of variance of initial eigenvalues	Cumulative per cent
1	7.900	43.888	43.888	7.900	43.888	43.888
2	2.177	12.092	55.980	2.177	12.092	55.980
3	1.710	9.501	65.481	1.710	9.501	65.481
4	1.323	7.352	72.833	1.323	7.352	72.833
5	.851	4.730	77.563	.851	4.730	77.563
6	.479	2.660	80.224			
7	.438	2.435	82.659			
8	.417	2.318	84.977			
9	.396	2.198	87.175			
10	.372	2.064	89.239			
11	.324	1.801	91.040			
12	.292	1.624	92.665			
13	.278	1.545	94.210			
14	.262	1.458	95.668			
15	.230	1.276	96.944			
16	.227	1.261	98.205			
17	.189	1.050	99.256			
18	.134	.744	100.000			

As can be seen from Table 4, the correspondence between the question items and the five common factors in the rotated component matrix table is the same as that set up, indicating that the questionnaire does not require further dimensionality reduction processing. The factor-loading coefficients corresponding to each question item under the common factors were all greater than 0.5, indicating that the questionnaire was set up reasonably.

**Table 4:** Component matrix after rotation.

Items	1	2	3	4	5
KT1	.245	**.761**	.051	.187	.243
KT2	.222	**.833**	.053	.123	.154
KT3	.147	**.835**	.071	.041	.111
KT4	.187	**.830**	.075	.131	.151
PA1	.129	.028	**.877**	.075	.090
PA2	.150	.078	**.914**	.087	.112
PA3	.130	.100	**.904**	.082	.140
CA1	.282	.144	.144	**.813**	.178
CA2	.191	.212	.054	**.824**	.183
CA3	.229	.066	.082	**.859**	.211
RA1	.304	.313	.275	.266	**.673**
RA2	.365	.219	.127	.228	**.738**
RA3	.183	.254	.137	.254	**.807**
DT1	**.751**	.153	.167	.229	.227
DT2	**.733**	.166	.042	.240	.172
DT3	**.727**	.237	.150	.187	.288
DT4	**.747**	.226	.139	.209	.123
DT5	**.813**	.178	.122	.070	.078

Factor analysis showed that knowledge transfer corresponded to Factor 2, perceived ability corresponded to Factor 3, capture ability corresponded to Factor 4, reconstructive ability corresponded to Factor 5, and digital transformation corresponded to Factor 1. The initial test validity was good, and we can continue with the validation factor analysis, calculation of convergent validity, and discriminant validity.

Confirmatory Factor Analysis (CFA) is often used to conduct a validity test of the scale, which can reflect the relationship between the items and variables. In this study, the factor loadings of each item, AVE values, and component reliability (CR) of the variables were obtained using confirmatory factor analysis to judge whether the validity of the questionnaire was qualified. The results of the validation factor analysis are shown in Table 5.

**Table 5:** Confirmatory factor analysis.

Variable	Item	Factor loadings	AVE	CR
Knowledge transfer	KT1	0.761	0.665	0.888
	KT2	0.833		
	KT3	0.835		
	KT4	0.830		
Perceived ability	PA1	0.877	0.807	0.926
	PA2	0.914		
	PA3	0.904		
Capture ability	CA1	0.813	0.693	0.871
	CA2	0.824		
	CA3	0.859		
Reconstructive ability	RA1	0.673	0.550	0.785
	RA2	0.738		
	RA3	0.807		
Digital Transformation	DT1	0.751	0.570	0.869
	DT2	0.733		
	DT3	0.727		
	DT4	0.747		
	DT5	0.813		

Note: *** represents p-value less than 0.001

As can be seen from the above table, the item factor loadings of the five variables, namely knowledge transfer, perceived ability, capture ability, reconstructive ability, and digital transformation, were all greater than 0.6, the combined reliability was greater than 0.7, and the average variance extracted (AVE) was greater than 0.5, which indicates that the convergent validity of the questionnaire in this study meets the requirements.

In addition, we need to use the scholars’ discriminant method of discriminant validity to further judge the discriminant validity of the six variables involved in this study. The AVE average variance extracted was greater than the correlation coefficient between variables. The questionnaire is considered to have good discriminant validity when the open root sign value of the AVE is greater than the correlation coefficient. As shown in Table 6.

**Table 6:** Distinguish between validity and correlation coefficients.

Variable	KT	PA	CA	RA	DT
**KT**	**0.818**				
**PA**	0.212	**0.884**			
**CA**	0.383	0.257	**0.850**		
**RA**	0.560	0.392	0.584	**0.831**	
**DT**	0.510	0.344	0.542	0.629	**0.782**

From the above table, it can be concluded that the discriminant validity between the study variables was good. Based on the results of the above analyses, the questionnaire used in this study passed reliability and validity tests. The questionnaire data can be used to construct structural equation modeling and verify the research hypotheses presented in this paper.

### 4.3 Correlation analysis

This study measured the correlation between two variables based on Pearson’s correlation coefficient and analyzed the data samples for correlation. The Pearson’s correlation coefficient was between -1 and 1. If the correlation coefficient between two variables is greater than zero, it indicates a positive correlation; if it is less than zero, it indicates a negative correlation. The closer the absolute value of the correlation coefficient is to 1, the stronger the correlation between the two variables. In this study, a correlation test of the relationship between the five sub-variables involved was carried out using SPSS24.0, and the test results are shown in Table 7.

**Table 7:** Correlation analysis results of each variable.

Variable	KT	PA	CA	RA	DT
**KT**	**1**				
**PA**	0.212**	**1**			
**CA**	0.383**	0.257**	**1**		
**RA**	0.560**	0.392**	0.584**	**1**	
**DT**	0.510**	0.344**	0.542**	0.629**	**1**

The results indicate a significant positive correlation between knowledge transfer and digital transformation, with a Pearson correlation coefficient of 0.510, thus preliminarily verifying hypothesis H1. Knowledge transfer enables enterprises to acquire relevant technical and management knowledge, enhance their knowledge stock and structure, and facilitate digital transformation. Additionally, knowledge transfer shows significant positive correlations with the dynamic capability dimensions of perceived ability (0.212), capture ability (0.383), and reconstructive ability (0.560), validating hypotheses H2, H3, and H4. The mediating variables of perceived ability (0.344), capture ability (0.542), and reconstructive ability (0.629) also correlated positively with digital transformation, thereby preliminarily validating hypotheses H5, H6, and H7. Perceived ability allows firms to identify digital opportunities and threats; capture ability enables timely responses to these insights; and reconstructive ability facilitates ongoing innovation and effective change management.

### 4.4 Analysis of structural equation modelling

#### 4.4.1 Modeling analysis

**Fig 2:**
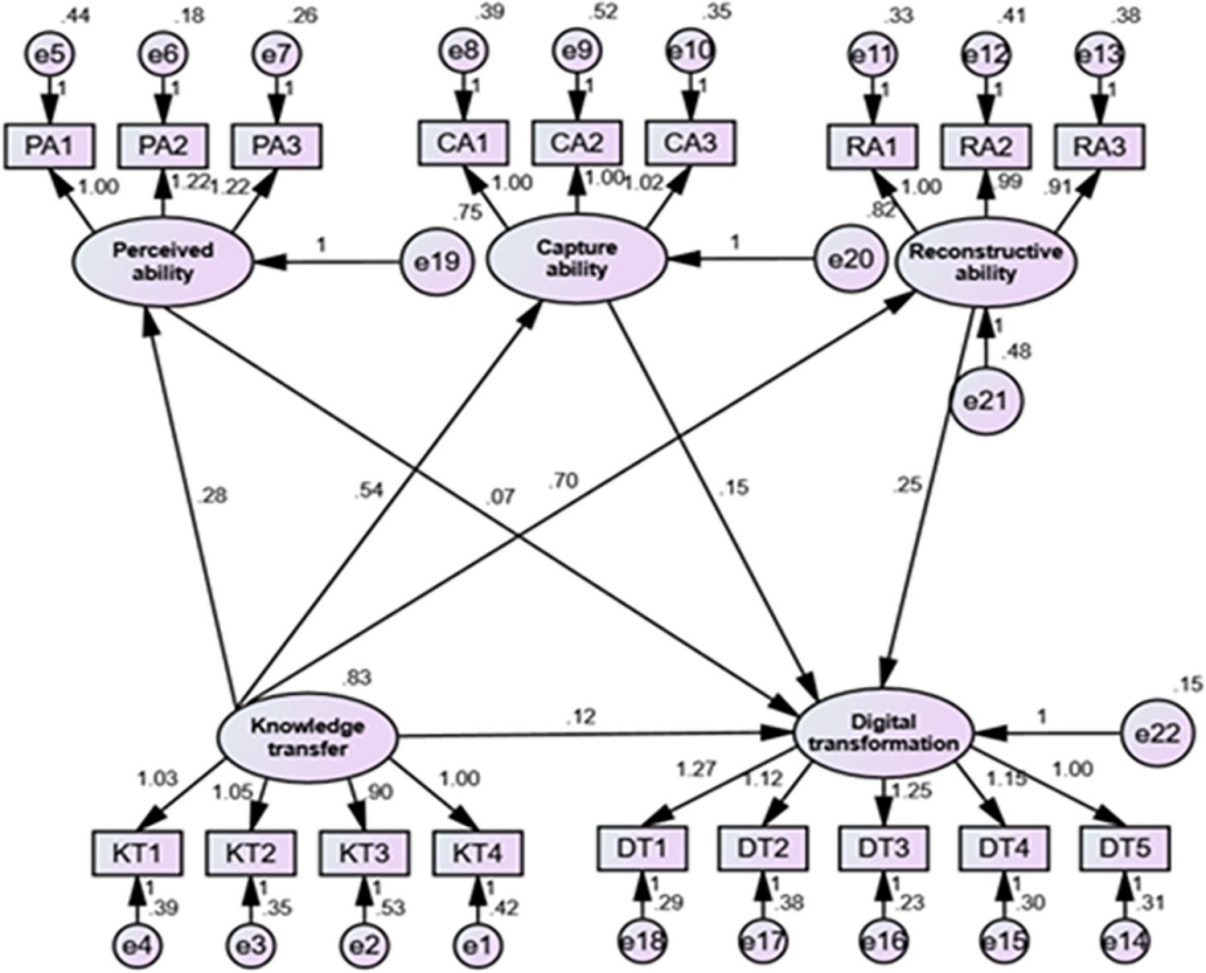
Structural equation model.

In this study, the proposed hypotheses were tested through a structural equation modeling approach using AMOS software. First, based on the hypothetical model, a structural equation modeling diagram was drawn using AMOS software, as shown in Fig 2.

#### 4.4.2 Model fit test

Before verifying whether the paths and assumptions are valid, the model type fit should be verified, which means that the structural equation model is reasonable and able to carry out the next step of hypothesis testing; if not qualified, it must be recorded. The specific model fits are listed in Table 8.

**Table 8:** Structural equation model fitting results.

Common indicators	X^2^	df	X^2^/df	GFI	RMSEA	RMR	CFI	NFI
**Criteria for judgement**	-	-	<3	>0.9	<0.08	<0.05	>0.9	>0.9
**Modelling**	129.174	125	1.033	0.938	0.013	0.041	0.998	0.949

As can be seen from the results of the model test, all the indicators reached the model fit standard: the X^2^/df value is 1.033, which is much smaller than 3; the RMSEA value is 0.013, which is also smaller than 0.08; the RMR value is 0.041, which is smaller than 0.05; the GFI value is 0.938, the CFI value is 0.998, and the NFI value is 0.949, which is larger than 0.9. All indicators met the standard requirements, indicating that the structural validity of the sample scale was very good, the overall model fit was high, and its fitting results were acceptable.

#### 4.4.3 Test of model hypothesis

As can be seen from the above table, the model fitness indicators all meet the recommended standards. The model as a whole has good structural validity and can be tested for hypotheses; the path coefficients between the variables are shown in Table 9 below.

**Table 9:** Path coefficient test table.

Pathway relationship	Standardized coefficient	Standard error	T-value	P-value	Conclusion
Knowledge transfer → Perceived ability	0.282	0.077	3.651	***	Support
Knowledge transfer → Capture ability	0.478	0.090	6.029	***	Support
Knowledge transfer → Reconstructive ability	0.678	0.082	8.611	***	Support
Knowledge transfer → digital transformation	0.195	0.057	2.169	0.001	Support
Perceived ability → digital transformation	0.115	0.041	1.804	0.036	Support
Capture ability → digital transformation	0.269	0.043	3.469	***	Support
Reconstructive ability → digital transformation	0.404	0.064	3.854	***	Support

Note: *** represents significance at p less than 0.001 significant

The path coefficient test revealed that knowledge transfer significantly positively affected perceived ability (0.282, p<0.001), capture ability (0.478, p<0.001), and reconstructive ability (0.678, p<0.001). Additionally, knowledge transfer had a positive effect on digital transformation, with a path coefficient of 0.195 (p<0.05). Furthermore, perceived ability (0.115, p<0.05), capture ability (0.269, p<0.001), and reconstructive ability (0.404, p<0.001) positively influenced digital transformation. Thus, hypotheses H1, H2, H3, H4, H5, H6, and H7 were validated and supported.

#### 4.4.4 Mediation effect test

This study applies a sample size of 2000 using Bootstrap sampling to generate confidence intervals at a 95% confidence level. This verifies the mediation effects by using the Bias-Corrected Bootstrap method. A significant direct effect was confirmed if the direct effect confidence interval did not include 0, whereas a significant mediation effect was validated if the indirect effect confidence interval did not include 0. If the direct effect includes 0 but the indirect effect does not, the variable is considered to be a full mediator. The specific mediating effects of each variable and its mediation relationship test are presented in Table 10.

**Table 10:** Mediation effect test.

Type of effect	Path	Effect value	Bootstrap Confidence Interval	
			Lower	Upper
Total effect	Knowledge transfer → digital transformation	0.485	0.372	0.598
Direct effect	Knowledge transfer → digital transformation	0.2101	0.086	0.317
Indirect effect	Knowledge transfer → capture ability → digital transformation	0.022	0.001	0.054
	Knowledge transfer → capture ability → digital transformation	0.088	0.037	0.157
	Knowledge transfer → reconstructive ability → digital transformation	0.174	0.102	0.264

According to the table above, the indirect and direct effects of the three paths do not contain 0. Therefore, it can be concluded that perception capability partially mediates knowledge transfer and digital transformation; capture ability partially mediates knowledge transfer and digital transformation; and reconstructive ability partially mediates knowledge transfer and digital transformation. Thus, hypotheses H8, H9, and H10 were validated and supported.

## 5 Results and discussion

### 5.1 Results

1. Knowledge transfer significantly and positively affects enterprises’ digital transformation. Enterprises acquire digital transformation-related technical knowledge and management practice knowledge through knowledge transfer behaviors, which increases the enterprise knowledge stock, changes the enterprise knowledge structure, applies the knowledge to the enterprise, compensates for the lack of experience in digital transformation practice, and promotes the digital transformation process of the enterprise.
2. Knowledge transfer significantly and positively affects an enterprise’s dynamic capabilities. Knowledge transfer provides information on technology, management and market for enterprises to improve their perceived capabilities, enabling them to identify and perceive new technological knowledge more clearly and accurately; at the same time, the act of knowledge transfer applies the acquired knowledge to enterprises, promotes the integration of knowledge, and improves the ability of enterprises to capture digital opportunities; in addition, knowledge transfer integrates heterogeneous knowledge with the enterprise’s original knowledge to favors the resetting of enterprise resources and improves the reconstruction ability of the enterprise.
3. Dynamic capabilities significantly and positively affect enterprises’ digital transformation. Perceived ability enables enterprises to filter out a large amount of effective information in the digital context, thereby reducing the information search cost of enterprises. In addition, capture ability enables firms to respond quickly to perceived digital opportunities. Finally, reconstructive ability enables enterprises to strengthen the underlying design, make organizational structure changes, and form an enterprise culture of digital knowledge learning and application.
4. Dynamic capabilities partially mediate knowledge transfer and digital transformation. Knowledge transfer enhances an enterprise’s perceived ability to anticipate and identify digital opportunities while meeting customer needs, which supports the enterprise’s digital transformation and drives its digital transformation process. In knowledge transfer, the frequent acquisition and application of digital technologies and management knowledge improve the capture capabilities of the firm and enhance digital transformation. With the continued application of digital technologies and management knowledge, enterprises have made strategic changes to adapt to digital transformation, improve their reconstructive ability, and further drive their digital transformation.

### 5.2 Discussion

#### 5.2.1 Knowledge transfer and digital transformation

Knowledge transfer significantly and positively affects enterprises’ digital transformation. This finding aligns with research conducted by Linde [14], indicating that acquiring and implementing knowledge related to digital technology and operations management can elevate the extent and quality of digital transformation within enterprises. It facilitates the increased utilization of digital technology across various facets of operations management, marketing services, and manufacturing; boosts employees’ grasp of digital transformation; and supports efficient digital process management within enterprises.

Further literature review shows that the digital transformation of enterprises is a complex process covering new product development, organizational change, business process improvement, management concept update, and business model innovation, reflecting a profound change at the strategic level. The complexity of this process requires enterprises to attach great importance to the acquisition and application of knowledge during the transformation process to adapt to the rapidly evolving digital era. Traditional business models and ways of thinking often fail to meet the requirements of the current market environment [3, 11, 15]. Therefore, enterprises must prioritize acquiring and leveraging knowledge in digitalization, recognizing that traditional business models and practices may be inadequate in the digital era.

Compared with previous studies, many scholars have realized the critical role of knowledge in digital transformation, addressing how knowledge can drive innovation and resilience in firms [12, 15, 24]. Kraus et al. pointed out that the enhancement of digital technology capabilities is closely related to the overall strategy of the firm, and knowledge transfer plays a mediating role in this process [38]. These studies corroborate our findings, suggesting that in today’s business environment, knowledge transfer is not only an enabler of digital transformation but also a foundation for achieving long-term competitive advantage for firms.

In conclusion, this study deepens our understanding of the importance of knowledge transfer in digital transformation strategies by empirically supporting the impact of knowledge transfer on corporate digital transformation. This not only provides a foundation for the construction of the theory but also practical guidelines for enterprises in the process of implementing digital transformation. Only by continuously strengthening knowledge transfer can enterprises occupy a favorable position in the changes of the digital economy era to successfully achieve the goal of digital transformation.

#### 5.2.2 Knowledge transfer and dynamic capabilities

Knowledge transfer significantly and positively affects an enterprise’s dynamic capabilities (perceived, capture, and reconstructive ability). This is in line with the findings of most scholars on teaching style and learning engagement of university teachers [3, 28, 31]. They emphasized the impact of teachers’ teaching styles on learning engagement, reflecting the importance of knowledge transfer in educational contexts. Extending this theory to the corporate environment, it can be seen that knowledge transfer has an equally positive impact on perceived, captured and restructuring capabilities. Knowledge transfer can significantly increase a firm’s knowledge stock, which forms the basis for renewing and upgrading organizational capabilities.

A firm’s knowledge structure, that is, the way its knowledge is composed and organized, is directly determined by its knowledge stock. A good knowledge structure helps firms perceive and capture market opportunities and threats more effectively, and provides a rational approach to resource reallocation [25]. As mentioned by Peng et al., organizations must have flexible perception mechanisms to identify potential opportunities and risks in a timely manner in a rapidly changing digital environment [16].

By acquiring knowledge and utilizing it in the knowledge transfer process, enterprises have heterogeneous digital knowledge that they originally lacked, which provides rich information for them to perceive changes in the digital environment and enhance their perceived ability, integrate and reconfigure the acquired knowledge with the organization’s original knowledge, and apply it to the enterprise’s digitalization practices. This improves workflows and practices, enhances the enterprise’s capture capabilities, promotes change and reconstruction of the enterprise, and strengthens its reconstructive ability [30].

In summary, the positive impact of knowledge transfer is manifested at multiple levels: it not only improves the perceived and captured capabilities of the enterprise but also fundamentally contributes to the reconstructive ability of the enterprise. The results of this research, combined with existing theoretical foundations, provide strong support for companies to develop strategies and tactics for digital transformation.

#### 5.2.3 Dynamic capabilities and digital transformation

Dynamic capabilities (perceived ability, capture ability, and reconstructive ability) exhibit significant positive impacts on digital transformation. This outcome aligns with the consensus among the majority of scholars both domestically and internationally [3, 5, 30]. Studies have shown that perceived ability enables enterprises to identify potential digital opportunities and threats in a rapidly evolving digital landscape, discern user needs, and adapt to environmental changes.

Perceived ability enables firms to identify potential digital opportunities and threats in complex and changing market environments. The perceived ability of firms not only helps them understand the changing needs of users, but also facilitates a keen grasp of industry trends. This capability enables firms to react faster and adjust their strategies to adapt to the requirements of a dynamic market. Capture ability, on the other hand, is key for firms to use the information gained from perceptions to act quickly. For example, Yang argued that firms with strong capture capabilities can effectively integrate knowledge resources and embed this knowledge into their daily operational processes [39]. This capability is essential to ensure that firms respond quickly to market demands and external changes, thereby avoiding lost opportunities. Reconfiguration capabilities form the basis of continuous innovation. By reconfiguring the organizational structure, firms can manage change more effectively and continue to innovate in process management and business models. Yin highlighted that reconfiguration capabilities enable firms to respond flexibly to changes in their internal and external environments, which in turn drives the success of their digital transformation [33].

In conjunction with previous research, it can be seen that perceiving, capturing, and reconstructing abilities form a dynamic capability framework that is essential for enterprises in the digital transformation process. This framework provides firms with competitive advantage in a rapidly changing digital economy. For example, Teece further elaborates on the concept of dynamic capabilities by highlighting the importance of organizations’ sustained competitiveness through sensing, capturing, and reconfiguring resources under changing market conditions [6].

In summary, perceived, captured, and reconstructive abilities play an important role in digital transformation. This not only matches existing research but also adds empirical support to the theoretical framework. By strengthening these capabilities, companies can adapt more effectively to changes in the digital environment and drive their own digital transformation and innovation. These findings provide an important reference point for companies when formulating their digital strategies, suggesting that the construction of dynamic capabilities is crucial in today’s competitive and ever-changing marketplaces.

#### 5.2.4 Mediating role of dynamic capabilities

Dynamic capabilities have a mediating role between knowledge transfer and digital transformation [15, 24, 38]. This finding is not only a deepening of the theoretical framework but is also in line with many national and international researchers, emphasizing the important role that dynamic capabilities play in this process.

First, the digital transformation process requires firms to actively update their knowledge resources. Knowledge transfer provides an important knowledge and information base for firms to build dynamic capabilities by facilitating knowledge sharing. As Li et al. point out, firms’ dynamic capabilities need to be built on rich knowledge resources to quickly identify and adapt to market changes [18]. In this context, perceived ability helps firms gain insight into the implications of advanced digital technologies and best practices, enabling them to capture potential digital opportunities in a timely manner.

Second, dynamic capabilities not only enable enterprises to cope with complex market environments but also lead them to reconfigure their knowledge resources and redefine their business models. By effectively integrating and utilizing internal and external knowledge, firms can better understand the changes in customer needs. For example, Crupi highlighted that dynamic capabilities essentially facilitate firms’ digital transformation by sensing and capturing digital opportunities and reconfiguring organizational structures [30]. The study argues that knowledge transfer is the cornerstone of building dynamic capabilities, providing organizations with the necessary resources and support in an evolving business environment.

Additionally, dynamic capabilities can help firms mitigate the risks of digital transformation. Through enhanced sensing and capturing capabilities, enterprises can more clearly identify market trends and potential threats so that they can make targeted institutional and strategic adjustments [16]. This enables enterprises to respond more flexibly in the face of uncertainty, thus ensuring the success of digital transformation.

In summary, knowledge transfer drives enterprises’ digital transformation by enhancing their dynamic capabilities. This process provides a clear path for enterprises to enhance their sensing, capturing, and reconfiguring capabilities through effective knowledge transfer in order to successfully implement digital transformation. This finding is not only consistent with previous research findings but also provides a valuable reference for enterprises in the process of strategy formulation and implementation.

### 5.3 Research significance

#### 5.3.1 Theoretical significance

This study fills the research gap between knowledge transfer and dynamic capabilities, proposes a new theoretical model of ‘Knowledge Transfer-Dynamic Capabilities-Digital Transformation,’ and explores how knowledge transfer affects the effect of digital transformation by enhancing dynamic capabilities. Second, it further subdivided dynamic capabilities into sensing, capturing, and reconfiguring capabilities, thus enhancing the applicability of the theory in empirical research and helping to reveal how to effectively implement knowledge transfer in the process of digital transformation to improve the success rate. Second, it further subdivided dynamic capabilities into perception, capture, and reconstruction capabilities, which enhances the applicability of the theory in empirical research, thus helping to reveal how knowledge transfer can be effectively implemented to improve the success rate of transformation in the process of digital transformation. Finally, the study emphasizes that knowledge transfer is not only a way of acquiring resources but also an important source of motivation to enhance the dynamic capabilities of the enterprise, suggesting that academics should pay attention to the key role of knowledge resources in the adjustment of the enterprise’s strategy and promote the in-depth development of subsequent research in this field.

#### 5.3.2 Practical significance

First, this study provides strategic guidance for the digital transformation of enterprises, emphasizing that enterprises should proactively focus on the acquisition and application of external digital resources and enhance their dynamic capabilities through effective knowledge transfer to better cope with complex market environments. Second, the study points out the key role of knowledge transfer in constructing the dynamic capabilities of enterprises and suggests that enterprises should establish a good knowledge management mechanism in the process of digital transformation to promote internal and external knowledge flow and integration, thus improving innovation capability and market adaptability. Third, considering the flexibility of enterprises in acquiring knowledge for digital transformation, this study also provides suggestions for knowledge transfer methods and tools in practice, such as establishing external cooperation networks and using digital platforms for knowledge sharing, to help enterprises implement knowledge transfer more effectively and support their digital transformation process.

### 5.4 Limitations

This study recognizes the varying stages of digital transformation maturity among enterprises but does not differentiate between them, which could affect research outcomes. Organizations in the early stages may prioritize digital infrastructure over knowledge acquisition. This study uses a questionnaire-based empirical approach focused on enterprise perspectives, but it has limitations: a limited number of responses may impede the interpretability of the theoretical model and respondents’ subjective perceptions may not accurately reflect their actual situations.

## Supporting information

S1 Table Questionnaire. (DOCX)

S2 Table Knowledge transfer data. (CSV)
