# Supplementary material for: Examining the influence of knowledge transfer and dynamic capabilities on enterprise digital transformation
Source: PLoS One. 2024 Dec 16;19(12):e0311176. doi: 10.1371/journal.pone.0311176 (PMC11649124; doi:10.1371/journal.pone.0311176)
Supplement: S1 Table — (DOCX) [file pone.0311176.s001.docx]

## Questionnaire

Dear Sir/Madam:

Greetings! Thank you very much for taking time out of your busy schedule to complete this questionnaire. We hope to gain valuable input from you for a study that is being conducted. To you to issue this questionnaire is to collect research data, the questionnaire is filled out in an anonymous way, the answer is not right or wrong, you only need to fill in according to personal judgement and the actual situation of your company can rest assured that all of your information will be kept strictly confidential, I promise that I will not be disclosed to a third party or for other purposes, the results of the answer will not be on the individual and the company will not have any impact on the results of the answer, I hope that you will be able to give a true evaluation. Thank you again for your support and co-operation in this research, I wish you a happy life and a smooth work!

I. Basic Information

1. Number of years your company has been established

○1-5 years

○6-10 years

○11-20 years

○21 years or more

2. Number of employees

○50 or less

○51-100

○101-300

○301-500

○More than 500 people

3. Nature of your company

○State-owned and state-controlled enterprises and institutions

○Private and privately held enterprises

○Sino-foreign equity joint venture

○Other enterprises

4. Your position in your company

○Ordinary employee

○Manager

○Mid-level manager

○High-level manager

二、Knowledge Transfer Scale

| 二、Knowledge Transfer Scale | | | | | | |
| --- | --- | --- | --- | --- | --- | --- |
| Question number | Topic content | Very inconsistent | Less consistent | Generally | More consistent | Very consistent |
| 1 | By collaborating with other organisations or platforms, the midshipmen have gained a great deal of technical knowledge related to digital transformation. | 1 | 2 | 3 | 4 | 5 |
| 2 | By collaborating with other organisations or platforms, the midshipmen gained a great deal of operational management knowledge related to digital transformation. | 1 | 2 | 3 | 4 | 5 |
| 3 | Existing digital skills and capabilities are enhanced through collaboration with other organisations or platforms. | 1 | 2 | 3 | 4 | 5 |
| 4 | Already applying acquired knowledge related to digital transformation to products and services. | 1 | 2 | 3 | 4 | 5 |

三、Dynamic Capabilities Scale

| 三、Dynamic Capabilities Scale | | | | | | |
| --- | --- | --- | --- | --- | --- | --- |
| Question number | Topic content | Very inconsistent | Less consistent | Generally | More consistent | Very consistent |
| 1 | Firms can recognize the changes that new technological knowledge may bring to their company before their competitors do. | 1 | 2 | 3 | 4 | 5 |
| 2 | Businesses are able to have timely digital knowledge of their competitors. | 1 | 2 | 3 | 4 | 5 |
| 3 | Enterprises can quickly and accurately identify customer needs and changes in the market environment. | 1 | 2 | 3 | 4 | 5 |
| 4 | Enterprises can learn from other enterprises' advanced digital technology in a timely manner. | 1 | 2 | 3 | 4 | 5 |
| 5 | The ability to reallocate resources in a timely manner to cope with changes in the environment. |  |  |  |  |  |
| 6 | Enterprises are able to quickly apply new knowledge and technology to their production activities. |  |  |  |  |  |
| 7 | Enterprises are able to continuously update and iterate digital technology knowledge and management knowledge. |  |  |  |  |  |
| 8 | The organization encourages employees to learn about digital knowledge and technology. |  |  |  |  |  |
| 9 | Reward employees for applying digital knowledge to their daily activities. |  |  |  |  |  |

四、Digital Transformation Scale

| 四、Digital Transformation Scale | | | | | | |
| --- | --- | --- | --- | --- | --- | --- |
| Question number | Topic content | Very inconsistent | Less consistent | Generally | More consistent | Very consistent |
| 1 | Higher degree of enterprises applying digital technology to the service side of the marketplace. | 1 | 2 | 3 | 4 | 5 |
| 2 | The extent to which companies use digital technology in marketing services is high. | 1 | 2 | 3 | 4 | 5 |
| 3 | The extent to which organisations use digital technologies in manufacturing is high. | 1 | 2 | 3 | 4 | 5 |
| 4 | Employees understand how their performance relates to the organization’s digital objectives. | 1 | 2 | 3 | 4 | 5 |
| 5 | Companies have developed practices for managing digital processes. |  |  |  |  |  |
